# Supplementary material for: Impact of Isolation Procedures on the Development of a Preclinical Synovial Fibroblasts/Macrophages in an In Vitro Model of Osteoarthritis
Source: Biology (Basel). 2020 Dec 10;9(12):459. doi: 10.3390/biology9120459 (PMC7764613; doi:10.3390/biology9120459)
Supplement: Supplementary file 1 [file biology-09-00459-s001.pdf]

Table S1. Oligonucleotide primer sequences.

Target Gene	Primers (forward and reverse)	Product Size (bp)	GenBank Accession No.	Primer Efficiency (%)
**GAPDH**	CGGAGTCAACGGATTTGG<br>CCTGGAAGATGGTGATGG	218	NM_002046	99.2
**CD80**	TGGCTGGTCTTTCTCACTTC<br>TCAACAGAAACATTGTGACCAC	97	NM_005191	96.1
**CD86**	GGAACCAACACAATGGAGAG<br>AAACACGCTGGGCTTCATC	94	NM_006889	98.2
**CD163**	GCAATGGGGTGGACTTACCT<br>TCACCATGCTTCACTTCAACAC	126	NM_004244.5	99.1
**CD206**	TCGGGTTTATGGAGCAGGTG<br>TGAACGGGAATGCACAGGTT	121	NM_002438.3	96.6
**IL6**	TAGTGAGGAACAAGCCAGAG<br>GCGCAGAATGAGATGAGTTG	184	NM_000600	96.1
**TNFα**	AGCCCATGTTGTAGCAAACC<br>GGACCTGGGAGTAGATGAGGTA	149	NM_000594	96,2
**CCL3/MIP1α**	ACCAGTTCTCTGCATCACTT<br>CTTGGTTAGGAAGATGACACC	146	NM_002983.2	99.5
**CXCL10/IP10**	GGTGAGAAGAGATGTCTGAATCC<br>GTCCATCCTTGGAAGCACTGCA	102	NM_001565	95,6
**IL10**	CTTTAAGGGTTACCTGGGTTG<br>CTTGATGTCTGGGTCTTGG	100	NM_000572	99.7
**TGFβ1**	CAACAATTCCTGGCGATACCT<br>TAGTGAACCCGTTGATGTCC	196	NM_000660	98.3
**CCL18**	CTGCCTCGTCTATACCTCC<br>CGGCCTCTCTTGGTTAGGA	111	NM_002988.3	91.4

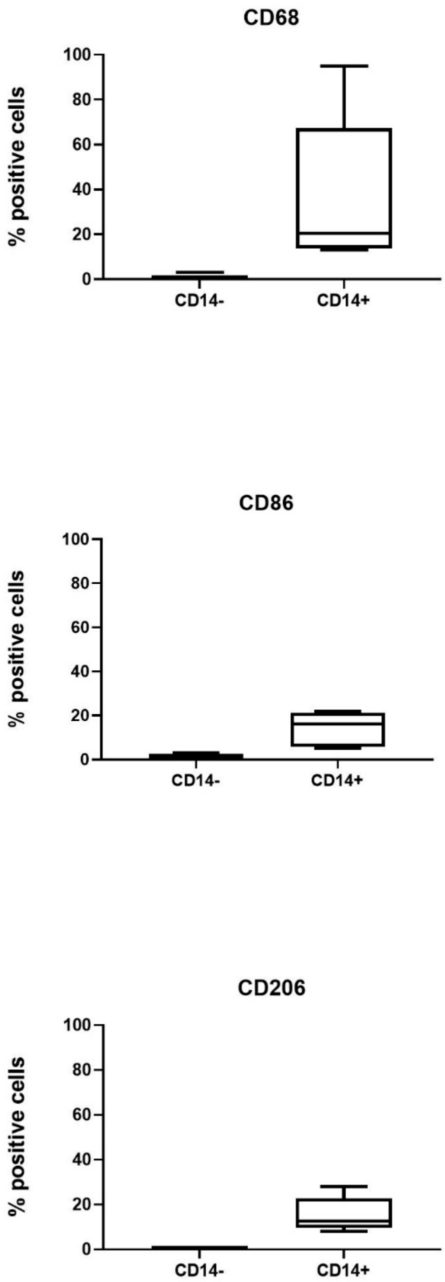

Figure S1. Flow cytometry evaluation of macrophages markers CD68, CD86 and CD206 evaluated both on CD14 positive (CD14+) and negative (CD14-) synoviocytes (*n* = 4). Data are expressed as percentage of positive cells and represented as box plot with median and minimum and maximum.

Publisher's Note: MDPI stays neutral with regard to jurisdictional claims in published maps and institutional affiliations.

© 2020 by the authors. Licensee MDPI, Basel, Switzerland. This article is an open access article distributed under the terms and conditions of the Creative Commons Attribution (CC BY) license (http://creativecommons.org/licenses/by/4.0/).
